# Nonpolarizing oxygen-redox capacity without O-O dimerization in Na_2_Mn_3_O_7_

**DOI:** 10.1038/s41467-020-20643-w

**Published:** 2021-01-27

**Authors:** Akihisa Tsuchimoto, Xiang-Mei Shi, Kosuke Kawai, Benoit Mortemard de Boisse, Jun Kikkawa, Daisuke Asakura, Masashi Okubo, Atsuo Yamada

**Affiliations:** 1 Department of Chemical System Engineering, School of Engineering, The University of Tokyo, Hongo 7-3-1, Bunkyo-ku Tokyo, 113-8656 Japan; 2 National Institute for Materials Science (NIMS), Tsukuba Ibaraki, 305-0044 Japan; 3 National Institute of Advanced Industrial Science and Technology (AIST), Umezono 1-1-1, Tsukuba Ibaraki, 305-8568 Japan; 4 Elements Strategy Initiative for Catalysts & Batteries (ESICB), Kyoto University, Nishikyo-ku Kyoto, 615-8245 Japan

**Keywords:** Batteries, Batteries

## Abstract

Reversibility of an electrode reaction is important for energy-efficient rechargeable batteries with a long battery life. Additional oxygen-redox reactions have become an intensive area of research to achieve a larger specific capacity of the positive electrode materials. However, most oxygen-redox electrodes exhibit a large voltage hysteresis >0.5 V upon charge/discharge, and hence possess unacceptably poor energy efficiency. The hysteresis is thought to originate from the formation of peroxide-like O_2_^2−^ dimers during the oxygen-redox reaction. Therefore, avoiding O-O dimer formation is an essential challenge to overcome. Here, we focus on Na_2-*x*_Mn_3_O_7_, which we recently identified to exhibit a large reversible oxygen-redox capacity with an extremely small polarization of 0.04 V. Using spectroscopic and magnetic measurements, the existence of stable O^−•^ was identified in Na_2-*x*_Mn_3_O_7_. Computations reveal that O^−•^ is thermodynamically favorable over the peroxide-like O_2_^2−^ dimer as a result of hole stabilization through a (σ + π) multiorbital Mn-O bond.

## Abstract (web summary)

Majority of oxygen-redox electrodes exhibit a large voltage hysteresis but its origin is not fully understood. Here, the authors use combined RIXS and magnetic measurements to provide insights into the origin of the typically large voltage hysteresis observed upon oxygen redox.

## Introduction

Lithium-ion batteries are presently the de facto standard power sources for portable electronic devices and electric vehicles due to their high energy density and efficiency relying on intercalation chemistry, whereby a host electrode material reversibly accommodates lithium ions without a large structural change^1–3^. As the reaction Gibbs energy for the oxidation of an electrode material (|Δ_r_*G*^ox^ | ) is almost the same as that for the reduction (|Δ_r_*G*^red^ | ), the charge/discharge processes of an intercalation electrode usually proceed with minimal energy loss. For any electrochemical energy storage devices, the use of reversible redox chemistry (|Δ_r_*G*^ox^ | ≈ |Δ_r_*G*^red^ | ) is a primary requisite to maximize their energy efficiency.

Lithium-rich transition metal oxides (Li_1+*x*_M_1-*x*_O_2_, M = transition metal) are promising large-capacity positive electrode materials for lithium-ion batteries, as they exhibit accumulative redox reactions of M and O^4–6^. However, the voltage profile of Li_1+*x*_M_1-*x*_O_2_ typically includes a large hysteresis during initial and subsequent charge/discharge cycles, in part due to structural changes such as cation migration and surface cation densification^7–9^. Although the oxygen-redox-active sodium counterpart Na_*x*_M_*y*_O_2_ can partly suppress cation migration due to the larger ionic size difference between Na and M, a large voltage hysteresis is still observed in many cases^10–18^. As energy efficiency is crucial for energy storage devices, the voltage hysteresis of oxygen-redox electrodes should be addressed for their practical application.

**Fig. 1:**
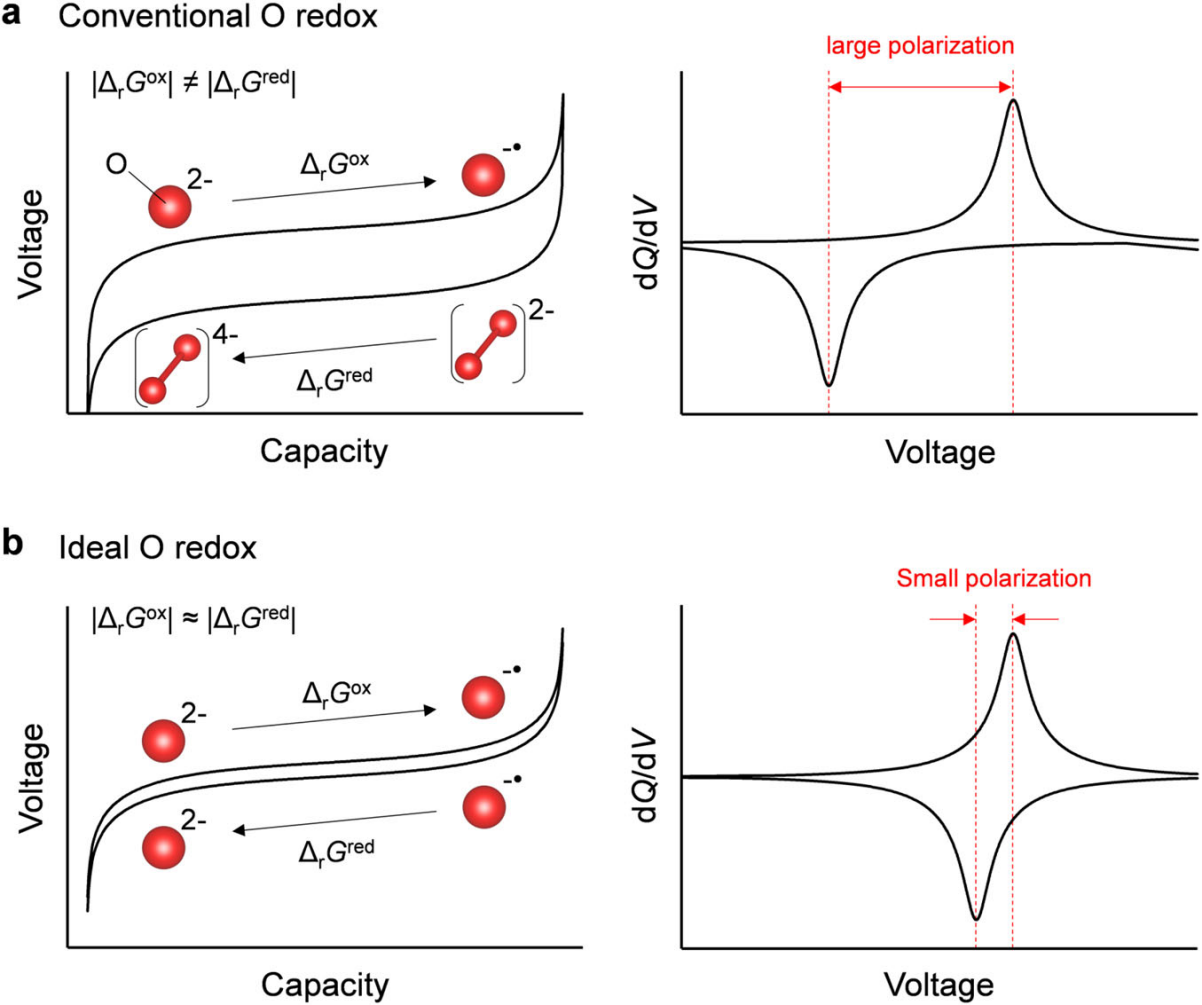
Polarizing and nonpolarizing oxygen-redox positive electrodes. Schematic illustration of charge/discharge curves and d*Q*/d*V* plots (*Q*: specific capacity, *V*: reaction voltage) for **a** conventional oxygen redox with large polarization (O^2−^/O_2_^2−^), and **b** ideal oxygen redox with small polarization (O^2−^/O^−•^). The red sphere denotes oxygen atom.

Although the mechanism of the oxygen-redox reaction is still under debate, it is generally accepted that nonbonding oxygen 2*p* states free from M-O σ hybridization are localized just below the Fermi level to contribute to oxygen oxidation^19–24^; however, the chemical state of oxidized oxygen remains controversial. Considering the large voltage hysteresis (|Δ_r_*G*^ox^ | ≠ |Δ_r_*G*^red^ | ), oxidized oxide ions (O^−•^) are believed to form stable peroxide-like O_2_^2−^ dimers upon charging. Upon subsequent discharging, the O_2_^2−^ dimer may be initially reduced to O_2_^4−^, and then decomposed to O^2−^ (Fig. 1a)^16,25–29^. Besides such thermodynamic hysteresis, there is an overlapping kinetic hysteresis arising from concentration overpotential when transition-metal migration and/or surface cation densification occur in parallel, making elucidation of the overall mechanisms difficult ^7–9^.

**Fig. 2:**
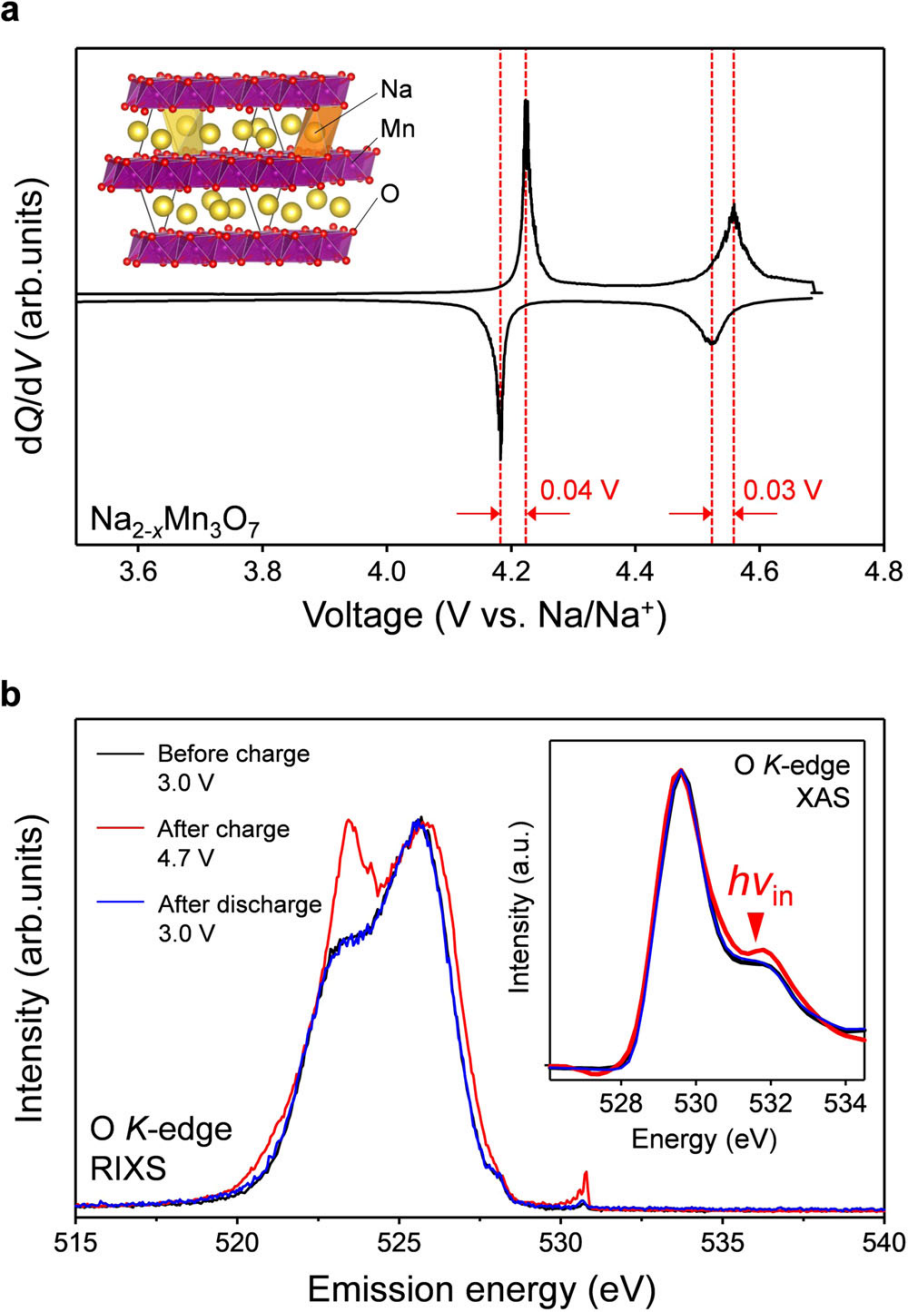
Non-polarizing oxygen-redox positive electrode Na_2_Mn_3_O_7_. **a** d*Q*/d*V* plot (*Q*: specific capacity, *V*: reaction voltage) of Na_2-*x*_Mn_3_O_7_ at C/20 during the second charge/discharge cycle between 3.0–4.7 V vs. Na/Na^+^. Inset shows the crystal structure of Na_2_Mn_3_O_7_ (yellow sphere: sodium, purple sphere: manganese, red sphere: oxygen). **b** O *K*-edge resonant inelastic X-ray scattering (RIXS) spectra for Na_2-*x*_Mn_3_O_7_ before the second charge (3.0 V, black line), after the second charge (4.7 V, red line), and after the second discharge (3.0 V, blue line) with excitation energy of 531.5 eV. Inset shows corresponding O *K*-edge X-ray absorption spectra (XAS).

In striking contrast to the large voltage hysteresis (>0.5 V) observed for most oxygen-redox electrodes, Na_2-*x*_Mn_3_O_7_ was very recently discovered to exhibit a highly reversible oxygen-redox capacity with negligible voltage hysteresis (<0.04 V)^30–33^. Hence, Na_2_Mn_3_O_7_ can serve as an excellent counterpart (Fig. 1b, |Δ_r_*G*^ox^ | ≈ |Δ_r_*G*^red^ | ) for insights into the origin of the typically large voltage hysteresis observed upon oxygen redox. Na_2_Mn_3_O_7_ possesses a layered structure comprising alternatively stacked Na and Mn slabs (Fig. 2a inset) with characteristic Mn vacancies (□) in $\sqrt 7 \times \sqrt 7$ in-plane ordering^34^. Density functional theory (DFT) calculations suggested that a localized oxygen 2*p* orbital along the Na-O-□ axis contributes to the oxygen-redox capacity^31^. While the nonpolarizing oxygen-redox capacity strongly implicates charge compensation from the reversible redox couple of O^2−^/O^−•^ (Fig. 1b) without any contribution from O_2_^2−^, no experimental evidence for the stable existence of O^−•^ has been identified for any oxygen-redox electrodes. Also important is unveiling how O^−•^ is stabilized in Na_2-*x*_Mn_3_O_7_.

Herein, evidences confirming the existence of O^−•^ as the dominant state in the highly reversible oxygen redox of Na_2-*x*_Mn_3_O_7_ are provided. After magnetic susceptibility measurements to confirm the major contribution of O^−•^, the thermodynamically favorable presence of O^−•^ over peroxide-like O_2_^2−^ in Na_2-*x*_Mn_3_O_7_ is substantiated via DFT calculations, and finally, the hole stabilization mechanism of O^−•^ is examined.

## Results

### Nonpolarizing oxygen redox in Na_2_Mn_3_O_7_

Na_2_Mn_3_O_7_ was synthesized from a solid-state reaction following a reported procedure^30,31^. The powder X-ray diffraction pattern for the resulting compound (Supplementary Fig. 1) is indexed to triclinic *P*$\overline 1$, confirming the successful synthesis of Na_2_Mn_3_O_7_. The selected-area electron diffraction (SAED) pattern (Supplementary Fig. 2a) reveals the ordered arrangement of Mn atoms and vacancies in the Mn slab ($\sqrt 7 \times \sqrt 7$ superlattice). The charge/discharge curves at a rate of C/20 show a reversible oxygen-redox capacity of approximately 70 mAh/g (Supplementary Fig. 3), which is consistent with a previous report^31^. Fig. 2a shows the d*Q*/d*V* plot for Na_2-*x*_Mn_3_O_7_ between 3.0 and 4.7 V vs. Na/Na^+^ during the second charge/discharge cycle. As the nominal valence state of Mn in Na_2_Mn_3_O_7_ is tetravalent (maximum valence of octahedral Mn), the redox center in Na_2-*x*_Mn_3_O_7_ is a priori attributable to oxygen; the Mn *L*-edge X-ray absorption spectra in our previous work confirmed the negligible contribution of Mn redox to the capacity^31^. Importantly, the d*Q*/d*V* peaks of the charge/discharge processes have an extremely small voltage hysteresis of merely 0.03–0.04 V, implicating the reversible redox reaction of oxygen (O^2−^/O^−•^) (Fig. 1b) without peroxide-like O_2_^2−^ formation (Fig. 1a).

O *K*-edge X-ray absorption spectra (Fig. 2b inset) show the emergence of a new absorption peak at 531.5 eV after the charge process, which corresponds to the excitation of an O 1 s core electron to an O 2p hole, as commonly observed for other oxygen-redox electrodes^35–38^. To monitor the detailed valence partial density of states (pDOS) of oxygen, resonant inelastic X-ray scattering (RIXS) spectra were measured using an incident photon of 531.5 eV. The emergence of a new emission peak at 523 eV in the RIXS spectrum for charged Na_2-*x*_Mn_3_O_7_ is typical for charged oxygen-redox electrodes^5,24,28,39,40^. Although O^−•^ formation is implicated by the small voltage hysteresis in the charge/discharge processes (Fig. 2a), the new inelastic scattering can be explained by either (1) energy loss from a π(Mn-O) → π^*^(Mn-O) transition (O^−•^ formation), or (2) energy loss from a σ(O-O) → σ^*^(O-O) transition (peroxide-like O_2_^2−^ formation)^40^. While observation of the new RIXS peak at 523 eV confirms the occurrence of the oxygen-redox reaction itself in Na_2-*x*_Mn_3_O_7_, but this evidence alone does not definitely identify the reaction mechanism, or the chemical state of the oxidized oxygen species.

### Magnetic elucidation of O^−^

**Fig. 3:**
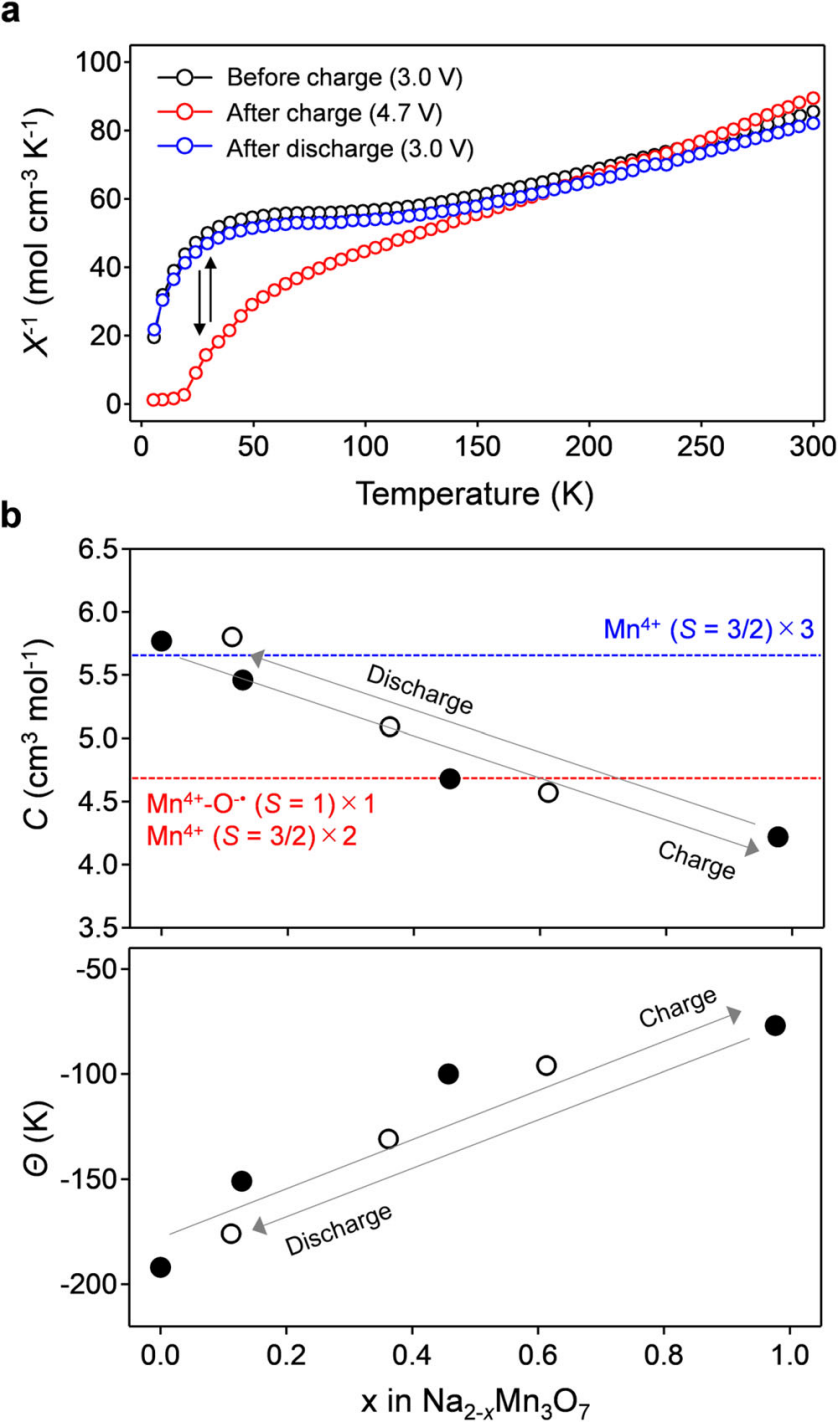
Magnetic elucidation of the existence of O^−^ in Na_2-*x*_Mn_3_O_7_. **a** The inverse of a magnetic susceptibility (*χ*) as a function of temperature, and **b** Curie constant (*C*) and Weiss temperature (*Θ*) for Na_2-*x*_Mn_3_O_7_ during the second cycle. Filled and empty circles correspond to data points for the charge and discharge processes, respectively.

To identify the oxidized species, we measured the magnetic susceptibility $\left( \chi \right)$ of Na_2-*x*_Mn_3_O_7_ with respect to its sensitivity to local coordination structures and electronic configurations. Figure 3a shows the temperature dependence of $\chi ^{{\mathrm{ - 1}}}$ during the second charge/discharge cycle. The $\chi ^{{\mathrm{ - 1}}}$ vs. *T* plot follows the Curie-Weiss law ($\chi = \frac{C}{{T - \Theta }}$, $C$ = Curie constant, $\Theta$ = Weiss temperature) at high temperature (> 200 K), providing the spin-state information of Mn and O in Na_2-*x*_Mn_3_O_7_. Because the ordered arrangement of Mn atoms and vacancies in the Mn slab is maintained after the charge process, as confirmed from the SAED pattern (Supplementary Fig. 2b), in-plane and out-of-plane Mn migrations do not occur in Na_2-*x*_Mn_3_O_7_. Therefore, the magnetic property changes are ascribed solely to the spin-state changes of Mn and O. Figure 3b shows the values of $C$ and $\Theta$ as a function of *x* in Na_2-*x*_Mn_3_O_7_. Before the charge process, the value of $C$ (5.77 cm^3^ K mol^−1^) agrees with the calculated value (5.63 cm^3^ K mol^−1^ for *g* = 2.0) for three Mn^4+^ (*S* = 3/2) in Na_2_Mn_3_O_7_. The negative $\Theta$ of −192 K indicates an in-plane antiferromagnetic interaction between Mn^4+^ cations^41^, which contradicts the prediction of ferromagnetic superexchange from the Goodenough-Kanamori rule^42^. This magnetic behavior is ascribed to the deviation of Mn-O-Mn bond angles from 90°, which most likely induces the antiferromagnetic superexchange; an antiferromagnetic *d*-*d* direct exchange interaction may also exist, as reported for Li_2_MnO_3_^43^.

The value of $C$ monotonically decreases to 4.22 cm^3^ K mol^−1^ upon charge, which is close to the calculated value (4.75 cm^3^ K mol^−1^ for *g* = 2.0) under a simple assumption that Mn^4+^-O^−•^ behaves as *S* = *S*_Mn_ + *S*_O_ = 1 (Zhang-Rice triplet)^44–46^. Note that the Zhang-Rice triplet in Na_2-*x*_Mn_3_O_7_ arises from π-type direct exchange while the Zhang-Rice singlet originally proposed for superconducting cupper oxides is generated via σ-type direct exchange^44^. Diamagnetic peroxide-like O_2_^2−^ formation, which should yield a constant *C*, is clearly ruled out by the observed decrease in *C*, as speculated from the very small hysteresis in the d*Q*/d*V* plot. The increase in $\Theta$ upon charge suggests the increment of ferromagnetic superexchange interaction between Mn spins through bridging O^−•^ over the effects of antiferromagnetic superexchange or *d*-*d* direct exchange. After discharge, both $C$ and $\Theta$ return to their initial values, supporting the reversibility of the charge/discharge processes without structural or electronic degradations. Overall, the magnetic measurements support the reversible oxygen-redox reaction of O^2−^/O^−•^ (Fig. 1b) in Na_2-*x*_Mn_3_O_7_.

### O^−•^ vs. O_2_^2−^

**Fig. 4:**
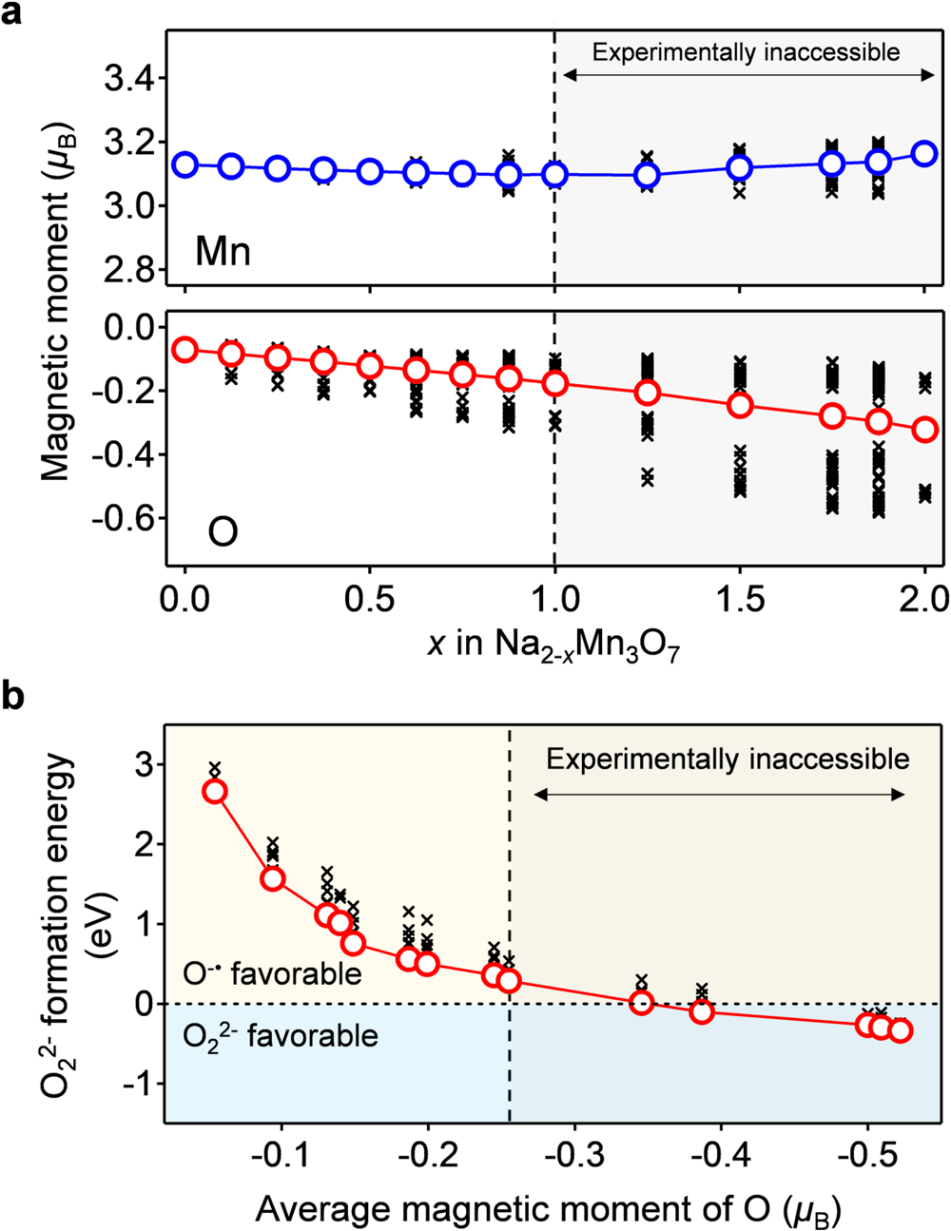
Theoretical derivation of stable existence of O^−•^ in Na_2-*x*_Mn_3_O_7_. **a** Calculated magnetic moments of Mn and O as a function of *x* in Na_2-*x*_Mn_3_O_7_, and **b** calculated formation energy of peroxide-like O_2_^2−^ as a function of the average magnetic moment (i.e., degree of oxidation) of O in Na_2-*x*_Mn_3_O_7_. For the plots of the magnetic moments, black crosses are a magnetic moment of each atom, while blue and red circles are average magnetic moments. For the plot of the formation energy, black crosses represent the formation energy of various O-O pairs, while red circles represent the lowest value at each desodiated state. Gray shaded area is experimentally inaccessible due to too high desodiation potential (see Supplementary Fig. S5).

**Fig. 5:**
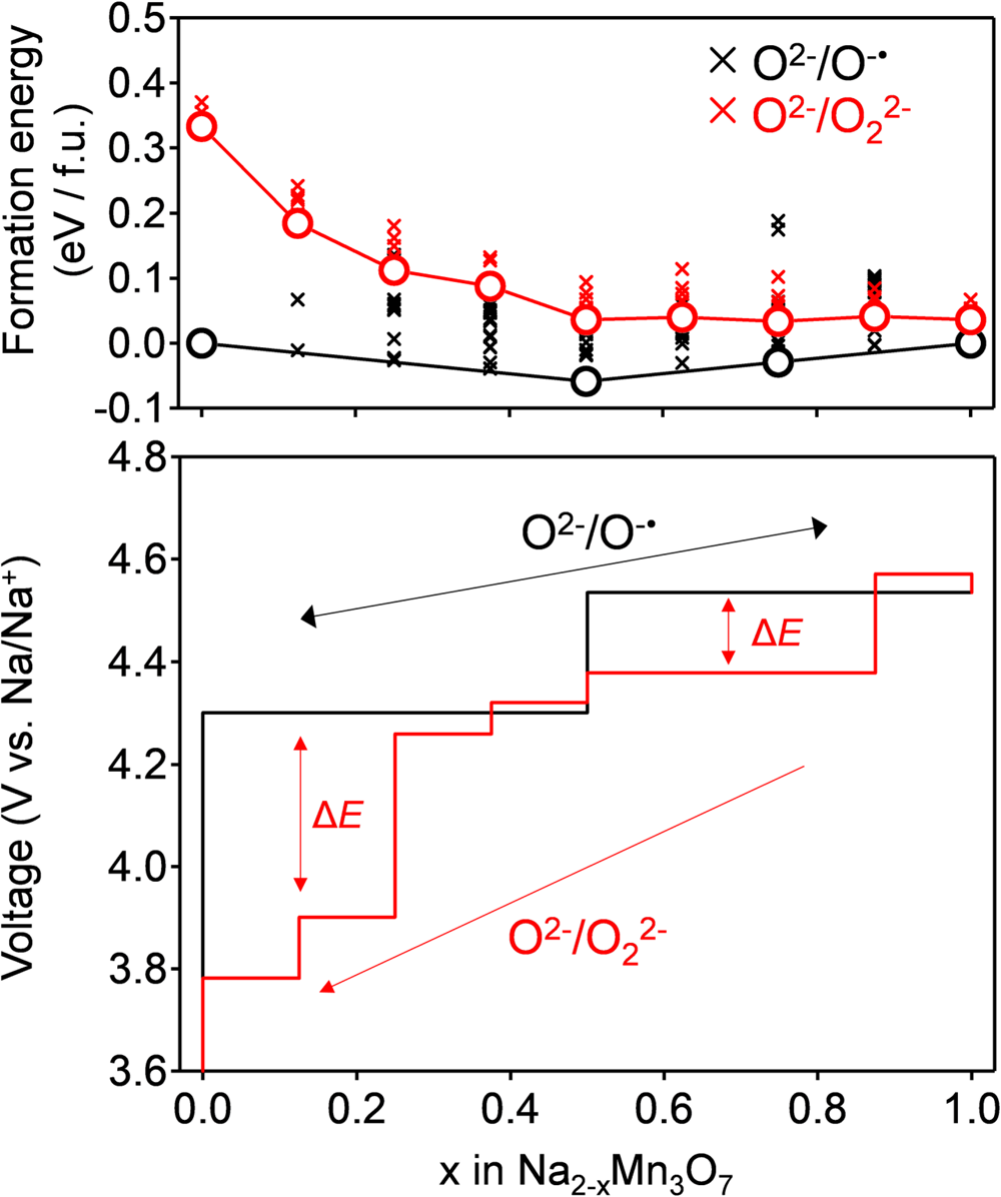
Predicted voltage hysteresis of Na_2-*x*_Mn_3_O_7_ with hypothetical peroxide-like O_2_^2−^ dimers. DFT calculated convex hull and voltage profile of Na_2-*x*_Mn_3_O_7_ with O^−•^ and with O_2_^2−^. The formation energies of both pristine structures and peroxide phases were calculated relative to pristine Na_2_Mn_3_O_7_ and NaMn_3_O_7_ phases. Black and red crosses in the convex hull are formation energies of Na_2-*x*_Mn_3_O_7_ with O^−•^ and with O_2_^2−^, respectively. Black and red circles are the lowest states at various desodiated states. The black solid line of the voltage profile represents the charge/discharge curves without O_2_^2−^ formation, while the red solid line represents the discharge curve for hypothetical Na_2-*x*_Mn_3_O_7_ with O_2_^2−^.

Why does Na_2-*x*_Mn_3_O_7_ specifically exhibit the reversible oxygen-redox reaction of O^2−^/O^−•^? To answer this question, we compared the thermodynamic stability of O^−•^ and peroxide-like O_2_^2−^ in Na_2-*x*_Mn_3_O_7_ using DFT calculations. Figure 4a shows the calculated magnetic moments of Mn and O in optimized structures without peroxide-like O_2_^2−^ (Supplementary Fig. 4a). For the experimentally accessible desodiation range (0 ≤ *x* ≤ 1 in Na_2-*x*_Mn_3_O_7_), a magnetic moment gradually emerges on the O atoms upon desodiation, which indicates O^−•^ formation. The slight decrease in the average magnetic moment of Mn is explained by the initiation of metal-to-ligand charge transfer (π back-donation) from the formation of a ligand hole (O^−•^)^40^. The calculated voltage profile under the O^−•^ formation model (Fig. 5 and Supplementary Fig. 5) accurately reproduces the experimentally observed voltage plateaus (4.30 and 4.53 V), further supporting O^−•^ formation in Na_2-*x*_Mn_3_O_7_.

The formation energy of peroxide-like O_2_^2−^ in Na_2-*x*_Mn_3_O_7_ was calculated for various O-O pairs, where calculated O-O bond lengths for the lowest energy structures are in the peroxide O_2_^2−^ range of 1.46-1.47 Å (Supplementary Fig. 4b)^47^. The average magnetic moment of the O-O dimers is small (-0.094(6) *μ*_B_), also suggesting the formation of peroxide-like O_2_^2−^ rather than superoxide-like O_2_^−^ when O-O dimerization occurs. The formation energy of O_2_^2−^ (Fig. 4b) was calculated as the difference between the total energies of Na_2-*x*_Mn_3_O_7_ with O^−•^ and with O_2_^2−^ as a function of the average magnetic moment of O (i.e., the degree of O oxidation). During the experimentally accessible charge process (0 ≤ *x* ≤ 1 in Na_2-*x*_Mn_3_O_7_), the formation energy of O_2_^2−^ is positive relative to that of O^−•^, indicating that Na_2-*x*_Mn_3_O_7_ with O^−•^ is thermodynamically favorable compared to Na_2-*x*_Mn_3_O_7_ with O_2_^2−^. Presumably, a hole on O^−•^ in Na_2-*x*_Mn_3_O_7_ is stabilized through a (σ + π) multiorbital Mn-O bond^40^, as demonstrated using a crystal orbital overlap populations (COOP) analysis in our previous works^31^. Note that, in parallel with a localized feature, the oxygen hole has an itinerant feature through the (σ + π) multiorbital interaction. Indeed, the experimentally observed value of *C* for Na_1_Mn_3_O_7_ (4.22 cm^3^ K mol^-1^) is slightly lower than that calculated for a localized model (4.75 cm^3^ K mol^−1^) (Fig. 3b).

Figure 4a also indicates that further oxygen oxidation leads to negative O_2_^2−^ formation energy (O_2_^2−^ favorable): the oxygen oxidation lowers the energy level of O 2*p* well below that of Mn *t*_2g_, which renders Mn-O interaction weak^48^. In the balance of competing stabilization mechanisms via (σ + π) multiorbital Mn-O bond versus peroxide O-O bond, the adequate oxygen-redox capacity (approximately 70 mAh/g, Supplementary Fig. 3) keeps the (σ + π) multiorbital stabilization dominant, making O^−•^ stable in Na_2-*x*_Mn_3_O_7_. It should also be emphasized that the calculated voltage profile for the sodiation of Na_2-*x*_Mn_3_O_7_ with O_2_^2−^ shows a voltage hysteresis of 0.3–0.5 V relative to the charge process of Na_2-*x*_Mn_3_O_7_ with O^−•^ (Fig. 5), which clearly contradicts the experimental observations. O_2_^2−^ formation is therefore inhibited in Na_2-*x*_Mn_3_O_7_, leading to an O^2−^/O^−•^ based reversible and nonpolarizing oxygen-redox capacity. Excessive oxidation of oxygen (e.g., *x* ≥ 1 in Na_2-*x*_Mn_3_O_7_), which drives O^−•^ to dimerization, is an absolute taboo for a nonpolarizing oxygen-redox capacity^48,49^. Based on this criterion, the oxygen-redox reaction should be employed as an auxiliary charge-compensation mechanism under a prudent control rather than as a main charge-compensation mechanism.

## Discussion

As recently demonstrated using O2-type layered oxides^23,50^, the suppression of cation migration is essential to mitigate the degradation of oxygen-redox cathodes. However, although cation migration should accelerate O-O dimer formation^16,20,51^, the suppression of cation migration alone is not enough to explain nonpolarizing oxygen-redox reaction. For example, P2- and P3-Na_2/3_Mg_*x*_Mn_1-*x*_O_2_ deliver large extra oxygen-redox capacities greater than 100 mAh/g with large polarization, where O-O dimers could be formed without cation migration^15,52^. This scenario, O-O dimerization without cation migration, is indeed predicted by the DFT calculations (Fig. 4). Correlation between cation migration and O-O dimerization involving thermodynamic and kinetic issues, and its influence on the voltage hysteresis is debatable, calling for further studies.

In summary, multiple experimental and computational pieces of evidence were identified to confirm the O^2−^/O^−•^ based reversible and nonpolarizing oxygen-redox reaction in Na_2-*x*_Mn_3_O_7_. The competitive O_2_^2−^ formation is energetically unfavorable when low-concentration O^−•^ (i.e., < Na_1_Mn_3_(O^−•^)_1_O_6_) is highly stabilized by a (σ + π) multiorbital Mn-O bond. To the best of our knowledge, this is the first experimental confirmation of the existence of O^−•^ in an oxygen-redox electrode. Considering the importance of energy efficiency, the exclusive use of O^2−^/O^−•^ as a redox couple is a primary requisite to utilize oxygen-redox electrodes in practical battery applications, identifying a crucial criterion for the development of efficient nonpolarizing oxygen-redox electrodes.

## Methods

### Electrochemistry

Na_2_Mn_3_O_7_ was synthesized by sintering the stoichiometric mixture of NaNO_3_ and MnCO_3_ at 600 °C under O_2_ flow for 4 h^30,31^. Electrochemical measurements were conducted using CR2032-type coin cells. Positive electrodes consisted of 80 wt% Na_2_Mn_3_O_7_, 10 wt% acetylene black, and 10 wt% polyvinylidene difluoride (PVDF), which were coated on Al foil using N-methylpyrrolidone (NMP) as the solvent. Sodium was used as the negative electrode, with 1.0 M NaPF_6_ in ethylene carbonate (EC)/diethyl carbonate (DEC) (1:1 v/v%) as the electrolyte. The cells were cycled at a charge/discharge rate of C/20.

### X-ray absorption/emission

Ex situ X-ray absorption spectroscopy (XAS) and resonant inelastic X-ray scattering (RIXS) measurements were performed on samples without exposure to air at BL07LSU of SPring‐8. A bulk‐sensitive partial fluorescence yield (PFY) mode was employed for O *K*‐edge XAS. Extended X-ray absorption fine structure was conducted at the BL-9C Beamline of the Photon Factroy, KEK, Japan. SAED patterns were recorded using an electron microscope (Titan Cubed, FEI Co.) operated at 80 kV after transferring the samples without exposure to air.

### Calculations

All structures were calculated using DFT, as implemented in the Vienna Ab Initio Simulation Package (VASP)^53,54^. The projector-augmented wave pseudopotential and a plane-wave basis set with an energy cut-off of 520 eV were used^55^. The generalized gradient approximation (GGA) with the Perdew-Burke-Ernzerhof functional describes the exchange-correlation energy^56,57^. To remove the self-interaction error, the Hubbard *U* correction was applied to the *d* electrons of Mn atoms (*U*_eff_ = 3.9 eV)^58,59^. The *k*-point was sampled on a 3 × 3 × 5 grid for all calculations. The Grimme scheme (DFT-D3) was applied to include van der Waals corrections^60^. Crystal structures were visualized with VESTA software ^61^.

We constructed a 2 × 2 × 1 supercell (Na_16-*i*_Mn_24_O_56_) to calculate a series of charged-structures of Na_2-*x*_Mn_3_O_7_ (0 < *x* ≤ 2). All possible cation orderings (Na^+^) were searched in the Supercell program, with the exclusion of symmetrical duplicates^62^. Considering the large number of configurations in the initial search (e.g., 1670 for Na_8_Mn_24_O_56_), we conducted a multi-step calculation of total energies to determine the stable cation ordering for each structure. First, we simply calculated the total energies without geometry optimization and selected the 100 lowest-energy configurations for each charged-structure. Second, we optimized both the lattice and atomic positions of the selected configurations under a force convergence of 0.03 eV Å^−1^, where the energy cut-off and number of *k* points were reduced to 400 eV and 1 × 1 × 3, respectively. At this point, we screened out the 20 lowest-energy configurations for each structure. Finally, we reconducted the geometry optimization with a more reliable energy cut-off (520 eV) and *k*-point mesh (3 × 3 × 5) under a force convergence of 0.01 eV Å^−1^, identifying all the lowest-energy structures in the charge process.

The convex hull was constructed to identify the stable phases among the determined structures, with the formation energies (at 0 K) calculated as:

1 $$\begin{array}{*{20}{c}} {E_{\mathrm{f}}({\mathrm{Na}}_{{\mathrm{2 - }}x}{\mathrm{Mn}}_3{\mathrm{O}}_7) = E({\mathrm{Na}}_{{\mathrm{2 - }}x}{\mathrm{Mn}}_3{\mathrm{O}}_7) - \frac{{2 - x}}{2}E({\mathrm{Na}}_2{\mathrm{Mn}}_3{\mathrm{O}}_7) - \frac{x}{2}E({\mathrm{Mn}}_3{\mathrm{O}}_7)} \end{array}$$

where *E*(Na_2-*x*_Mn_3_O_7_) is the total energy of Na_2-*x*_Mn_3_O_7_ at 0 ≤ *x* ≤ 2. The voltage profile was then evaluated^63^, with the average voltage of the reaction between two adjacent stable phases calculated as:

2 $$\begin{array}{*{20}{c}} {U(x_1,x_2) = - \frac{{E\left( {{\mathrm{Na}}_{2 - x_1}{\mathrm{Mn}}_3{\mathrm{O}}_7} \right) - E\left( {{\mathrm{Na}}_{2 - x_2}{\mathrm{Mn}}_3{\mathrm{O}}_7} \right) - \left( {x_2 - x_1} \right)\mu _{Na}}}{{\left( {x_2 - x_1} \right){\mathrm{F}}}}} \end{array}$$

where *μ*_Na_ is the chemical potential of Na metal and *F* is the Faraday constant. The representative structures are shown in Supplementary Fig. 4a.

The formation of peroxide-like O_2_^2−^ in Na_2-*x*_Mn_3_O_7_ was calculated by creating a short O-O bond in Na_16-*i*_Mn_24_O_56_, as shown in Supplementary Figure 4b. After the atomic positions were fully relaxed, no other significant structural change was found except for the O-O dimer. The formation energy of O_2_^2−^ was calculated by:

3 $$\begin{array}{*{20}{c}} {E_{\mathrm{f}}({\mathrm{O}}_2^{2 - },x) = E({\mathrm{O}}_2^{2 - },x) - E({\mathrm{Na}}_{2 - x}{\mathrm{Mn}}_3{\mathrm{O}}_7)} \end{array}$$

where *E*(O_2_^2−^, *x*) is the total energy of Na_2-*x*_Mn_3_O_7_ with peroxide-like O_2_^2-^. The voltage profile of the discharge process for Na_2-*x*_Mn_3_O_7_ with peroxide-like O_2_^2-^ was calculated based on the same cation orderings considered in the charge process.

## Supplementary information

Supplementary Information

Peer Review

## Data Availability

The whole datasets are available from the corresponding author on reasonable request.
