# Supplementary material for: Nonpolarizing oxygen-redox capacity without O-O dimerization in Na2Mn3O7
Source: Nat Commun. 2021 Jan 27;12:631. doi: 10.1038/s41467-020-20643-w (PMC7840947; doi:10.1038/s41467-020-20643-w)
Supplement: Supplementary file 1 — Supplementary Information [file 41467_2020_20643_MOESM1_ESM.pdf]

Nonpolarizing oxygen-redox capacity without O-O dimerization in $Na_2Mn_3O_7$

Akihisa Tsuchimoto,[1,#] Xiang-Mei Shi,[1,#] Kosuke Kawai,[1] Benoit Mortemard de Boisse,[1] Jun Kikkawa,[2] Daisuke Asakura,[3] Masashi Okubo,[1,4] Atsuo Yamada[1,4,*]

[1]Department of Chemical System Engineering, School of Engineering, The University of Tokyo, Hongo 7-3-1, Bunkyo-ku, Tokyo 113-8656, Japan

[2]National Institute for Materials Science (NIMS), Tsukuba, Ibaraki 305-0044, Japan

[3]National Institute of Advanced Industrial Science and Technology (AIST), Umezono 1-1-1, Tsukuba, Ibaraki 305-8568, Japan

[4]Elements Strategy Initiative for Catalysts & Batteries (ESICB), Kyoto University, Nishikyo-ku, Kyoto 615-8245, Japan

[*]yamada@chemsys.t.u-tokyo.ac.jp

[#]These authors contributed equally to this work.

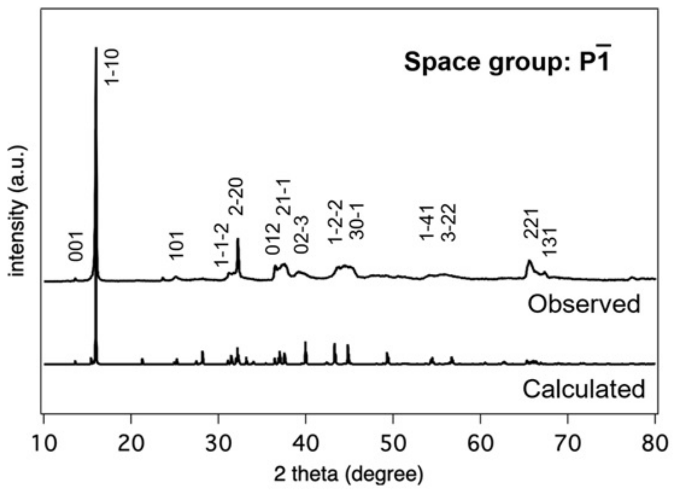

Supplementary Figure 1. Powder X-ray diffraction pattern for $Na_2Mn_3O_7$. A calculated pattern is also plotted for comparison.

(a) before charge

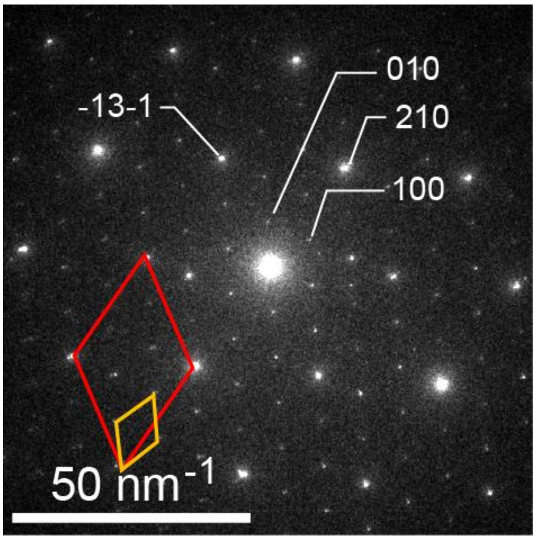

(b) after charge

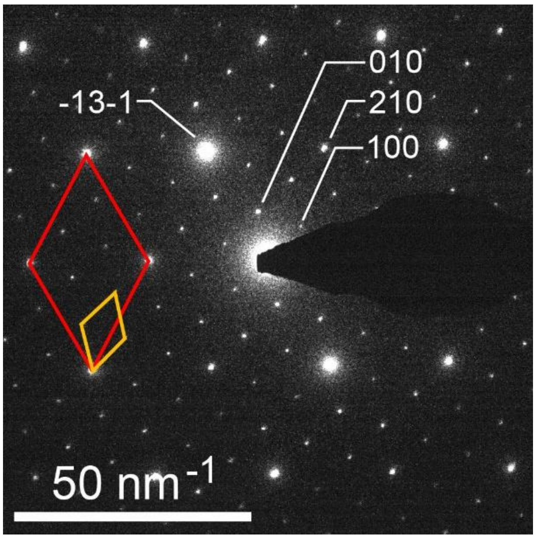

Supplementary Figure 2. Selected-area electron diffraction patterns for $Na_{2-x}Mn_3O_7$ (a) before charge and (b) after charge. The red cells are a typical trigonal sublattice of MnO_2, while the yellow cells are $\sqrt{7}\times\sqrt{7}$ superlattice from the ordered arrangement of Mn atoms and vacancies.

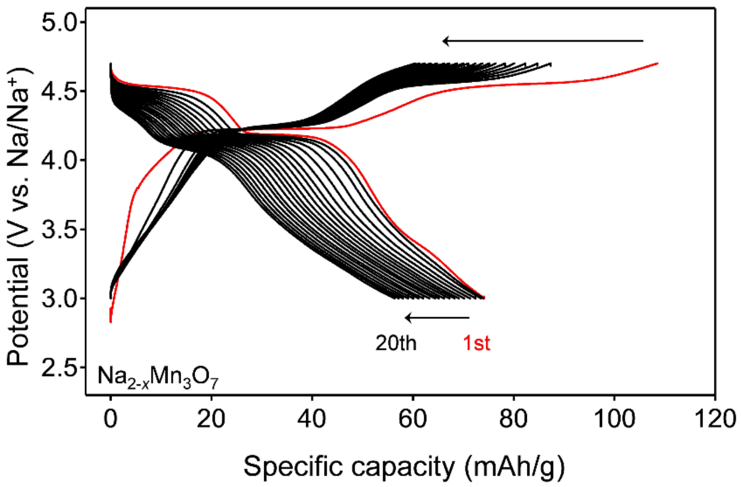

Supplementary Figure 3. Charge/discharge curves for $Na_{2-x}Mn_3O_7$ at a rate of C/20 between 3.0-4.7 V vs. Na/Na^+.

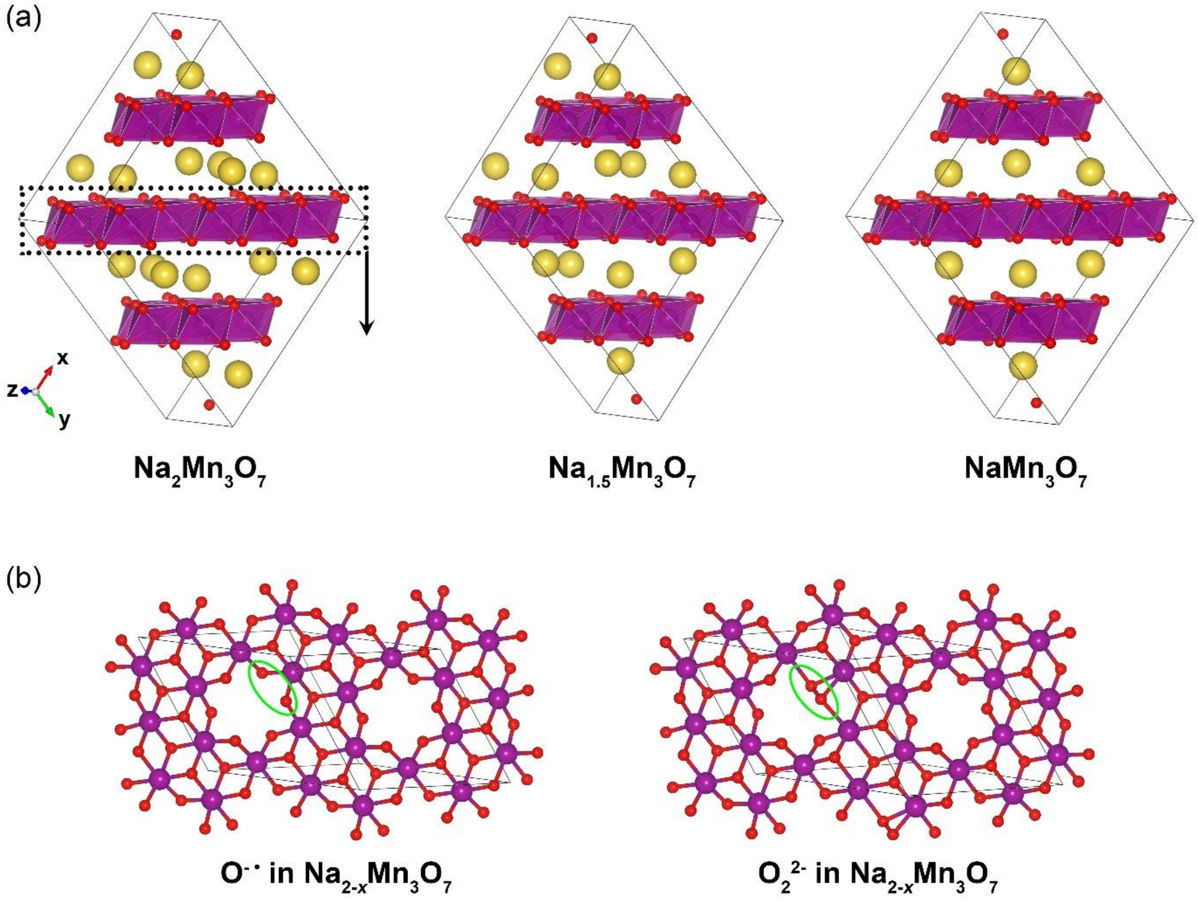

Supplementary Figure 4. (a) Optimized structures of $Na_2Mn_3O_7$, $Na_{1.5}Mn_3O_7$ and $NaMn_3O_7$, where purple, yellow and red spheres represent Mn, Na and O atoms, respectively. (b) Structural change before and after the peroxide-like O_2^{2-} formation in $Na_{2-x}Mn_3O_7$.

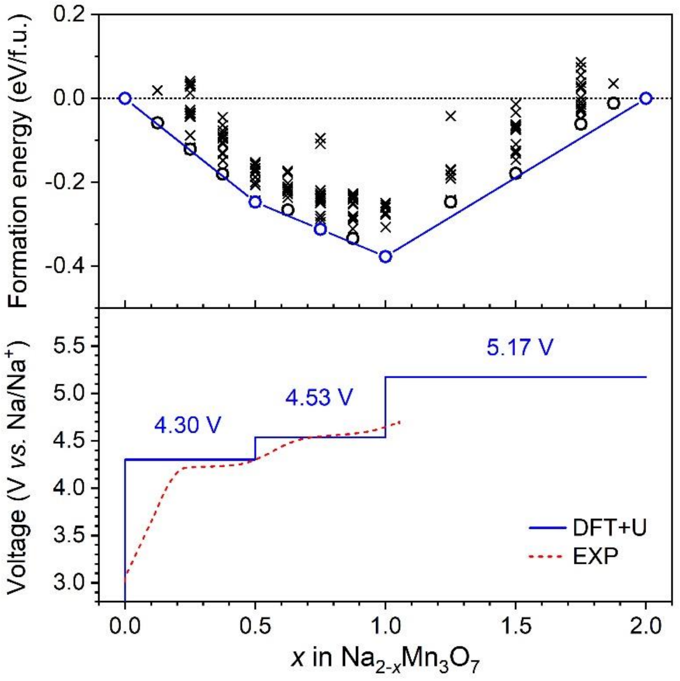

Supplementary Figure 5. DFT calculated convex hull and voltage profile of $Na_{2-x}Mn_3O_7$ without O-O dimerization. The formation energies were calculated relative to pristine $Na_2Mn_3O_7$ and Mn_3O_7 phases. Black crosses in the convex hull are formation energies, while black and blue circles are the lowest states and stable phases at various desodiated states, respectively. The experimental voltage profile (red dotted line) is also plotted for comparison.
