# Supplementary material for: Nonpolarizing oxygen-redox capacity without O-O dimerization in Na2Mn3O7
Source: Nat Commun. 2021 Jan 27;12:631. doi: 10.1038/s41467-020-20643-w (PMC7840947; doi:10.1038/s41467-020-20643-w)
Supplement: Supplementary file 2 — Peer Review [file 41467_2020_20643_MOESM2_ESM.pdf]

Reviewer #1 (Remarks to the Author):

The manuscript by Tsuchimoto et al reports on the oxygen redox upon Na deintercalation from $Na_2Mn_3O_7$ occurring through the O- radicals that allegedly explains low voltage hysteresis. The present manuscript is of little novelty compared to the previous publication of the same group in Adv. Energy Mater. (DOI: 10.1002/aenm.201800409). The previous article reports on oxygen redox in $Na_2Mn_3O_7$, provides very similar results of galvanostatic cycling, spectroscopic characterization and extensive DFT calculations. There is even one-to-one copy of the electron diffraction data from the Adv. Energy Mater. article (Fig. 3b) to the current manuscript (Fig. S2a), except of changing colors. The concept of (s + p) multiorbital bonds was also introduced in the recent publication of the same group (Energy Environ. Sci., DOI: 10.1039/c9ee04197d). All these were then compiled in this manuscript based on the only new data of magnetic susceptibility measurements, which indirectly support these "multiorbital bonds" by the lowered magnetic moment in the charged state. Neither further experimental support to this claim is provided nor the quantitative analysis of the charged and discharged structures is given. This manuscript provides little extra insight into the problem of voltage fade and should be published in a more specialized journal, preferably after getting more extensive experimental support on the structural and electronic changes in course of charge and discharge.

Reviewer #2 (Remarks to the Author):

This is an excellent piece of work about the redox mechanism of $Na_2Mn_3O_7$. While the concept of "anion redox" (mostly lattice oxygen redox) in battery cathodes has attracted tremendous amounts of attentions in recent years. The origin of this phenomenon is far from clear. The majority of these cathodes suffer from very large voltage hysteresis, leading to the poor energy efficiency. $Na_2Mn_3O_7$ belongs to a handful of examples (discovered so far) that show very small voltage hysteresis albeit dominant charge compensation from the lattice oxygen redox. In this study, by using combined RIXS and magnetic measurements, the authors undoubted confirmed that localized holes are created on the oxygen 2p states which is stabilized by forming potential spin singlet (or triplet if considering that Mn^{4+} in d3 configuration) between the single electrons on the oxygen 2p orbital and single electrons on the Mn t2g orbital. This electronic structure picture seems to be so far the most reasonable explanation for lattice oxygen redox in small or negative charge transfer gapped oxides. This model is probably also validated for many other cathode systems with later transition metal cations. I would expect this piece of work to stimulate further theoretical work in this direction. I would strongly recommend to publish this paper in Nature Communications.
With all that, I have a few questions/comments on some of the arguments made in this study:
1. The authors claim the formation of peroxide induces large voltage hysteresis. This statement contradicts many experiment observations. For instance, there is a very large voltage hysteresis in the P2 or P3-type $Na_{2/3}Mg_{1/3}Mn_{2/3}O_2$, yet the charge compensation mechanism is likely to be very similar to the model proposed here. There is no evidence of peroxide formation in these compounds. The absence of irreversible cation migration (to the Na^+ layer) seems to play a rather important role in the small voltage hysteresis.
2. There are two redox peaks, one at about 4.2 V and the other at about 4.5-4.6 V. This is different from most lattice oxygen redox systems where only one redox peak is observed. Is there any structure transition after the first voltage plateau. It would make this work more comprehensive if the authors could explain the correlation between the structure change and the oxygen redox potential change.
3. I would suggest to cite the following paper: CrO_2:A Self-Doped Double Exchange Ferromagnet. Phys. Rev. Lett. 80, 4305.
The original paper of Zhang-Rice singlet is on sigma anti-bonding (eg*) instead of the pi antibonding (t2g*).

Reviewer #3 (Remarks to the Author):

This paper describes the reversible anionic redox for $Na_2Mn_3O_7$ without O-O dimerization, and also provides interesting results for me. However, some points were not clear. Specific comments are described in the following section;

(1) Absence of O-O dimerization is explained as the origin of smaller voltage hysteresis. However, similar to this work, P3 $Na_{0.6}(Li_{0.2}Mn_{0.8})O_2$ also shows smaller polarization with possibly anionic redox (Goodenough, 2016 EES). For me, smaller voltage hysteresis is because of the Na chemistry, in which Mn mobility is low compared with the Li chemistry. Mn migration is often used to explain the origin of voltage hysteresis in the literature for the Li chemistry with anionic redox.

(2) In addition to the point (1), the concentration of holes formed in this sample is relatively low. A single hole is formed per three Mn ions and seven O ions, and I think that simply lower concentration of holes results in better stability and reversibility (Doublet, 2019 Nature Materials). Indeed, DFT results clearly show the stabilization of O-O dimers as increase in the concentration of holes. This fact inevitably results in that only small reversible capacity is obtained when "nonpolarizing" oxygen redox is used for battery applications.

(3) From the assumption of Zhang-Rice triplet formation, in other words, spin interaction between Mn-O occurs, and a hole must be localized in single oxygen. However, from the DFT data, holes seem to be delocalized, and therefore, spin interaction would be not simple as proposed by authors.

(4) In addition to the point (3), the model used for the magnetic moments seems to be underestimated compared with the experimentally observed values. Any thoughts?

Detailed responses to the review comments with point-to-point basis, including changes highlighted to the manuscript, are listed below.

Reviewer #1 (Remarks to the Author):

(1) The manuscript by Tsuchimoto et al reports on the oxygen redox upon Na deintercalation from $Na_2Mn_3O_7$ occurring through the O^- radicals that allegedly explains low voltage hysteresis. The present manuscript is of little novelty compared to the previous publication of the same group in Adv. Energy Mater. (DOI: 10.1002/aenm.201800409). The previous article reports on oxygen redox in $Na_2Mn_3O_7$, provides very similar results of galvanostatic cycling, spectroscopic characterization and extensive DFT calculations.

The concept of (s + p) multiorbital bonds was also introduced in the recent publication of the same group (Energy Environ. Sci., DOI: 10.1039/c9ee04197d). All these were then compiled in this manuscript based on the only new data of magnetic susceptibility measurements, which indirectly support these multiorbital bonds by the lowered magnetic moment in the charged state. Neither further experimental support to this claim is provided nor the quantitative analysis of the charged and discharged structures is given. This manuscript provides little extra insight into the problem of voltage fade and should be published in a more specialized journal, preferably after getting more extensive experimental support on the structural and electronic changes in course of charge and discharge.

Author reply: We dissent from these allegations without scrutiny. The reviewer falsely claims 'very similar results of galvanostatic cycling, spectroscopic characterization and extensive DFT calculations', and 'this manuscript based on only new data of magnetic susceptibility measurements'. However, the list of the contents of this manuscript below denies this false claim.

	Adv. Energy Mater.	This work
Electrochemistry	charge-discharge/CV	Spotlighting oxygen redox alone with emphasis on polarization issue using d*Q*/d*V* (Figure 2a)
Spectroscopy	X-ray absorption	X-ray emission (**Figure 2b**, **new results**)
Magnetic data	-	Curie constant/Weiss constant (**Figure 3**, **new results**)
Computation	DOS, COOP	Magnetic moment (**Figure 4a**, **new results**)<br>Dimerization energy (**Figure 4c**, **new results**)<br>Predicted voltage for dimers (**Figure 5**, **new results**)

These new results allow us to address a firm conclusion 'the electron hole formation (O^-/O^{2-}) is essential for nonpolarizing oxygen-redox reaction in $Na_2Mn_3O_7$', which was merely a speculation in our previous work published in *Adv. Energy Mater.* Also, the novelty and importance of this work are evident by referring the very positive comments from other two reviewers.

(2) There is even one-to-one copy of the electron diffraction data from the Adv. Energy Mater. article (Fig. 3b) to the current manuscript (Fig. S2a), except of changing colors.

Author reply: Thank you for the valuable comment. As explained in the main text, the SAED image of pristine $Na_2Mn_3O_7$ (original Fig. S2a, quoted from our previous paper in *Adv. Energy Mater.*) was placed just as a reference for comparison purpose with that of charged $Na_2Mn_3O_7$ (Fig. S2b). However, to avoid copyright infringement, we have replaced it with another SAED image of pristine $Na_2Mn_3O_7$.

Reviewer #2 (Remarks to the Author):

(1) This is an excellent piece of work about the redox mechanism of Na2Mn3O7. While the concept of "anion redox" (mostly lattice oxygen redox) in battery cathodes has attracted tremendous amounts of attentions in recent years. The origin of this phenomenon is far from clear. The majority of these cathodes suffer from very large voltage hysteresis, leading to the poor energy efficiency. Na2Mn3O7 belongs to a handful of examples (discovered so far) that show very small voltage hysteresis albeit dominant charge compensation from the lattice oxygen redox. In this study, by using combined RIXS and magnetic measurements, the authors undoubted confirmed that localized holes are created on the oxygen 2p states which is stabilized by forming potential spin singlet (or triplet if considering that Mn4+ in d3 configuration) between the single electrons on the oxygen 2p orbital and single electrons on the Mn t2g orbital. This electronic structure picture seems to be so far the most reasonable explanation for lattice oxygen redox in small or negative charge transfer gapped oxides. This model is probably also validated for many other cathode systems with later transition metal cations. I would expect this piece of work to stimulate further theoretical work in this direction. I would strongly recommend to publish this paper in Nature Communications.

Author reply: We highly appreciate the positive evaluation.

(2) The authors claim the formation of peroxide induces large voltage hysteresis. This statement contradicts many experiment observations. For instance, there is a very large voltage hysteresis in the P2 or P3-type $Na_{2/3}Mg_{1/3}Mn_{2/3}O_2$, yet the charge compensation mechanism is likely to be very similar to the model proposed here. There is no evidence of peroxide formation in these compounds. The absence of irreversible cation migration (to the Na^+ layer) seems to play a rather important role in the small voltage hysteresis. (There are two redox peaks, one at about 4.2 V and the other at about 4.5-4.6 V. This is different from most lattice oxygen redox systems where only one redox peak is observed. Is there any structure transition after the first voltage plateau. It would make this work more comprehensive if the authors could explain the correlation between the structure change and the oxygen redox potential change.

Author reply: As recently demonstrated by the research group led by Kisuk Kang (*Nature Mater.* **2020**, *19*, 419-427), the suppression of cation migration is essential for the cycle stability of oxygen-redox cathodes. However, we postulate that, although cation migration may accelerate O-O dimer formation (for example, *Nature Energy* **2020**, *5*, 777-785), the suppression of cation migration alone is not enough to explain nonpolarizing oxygen-redox reaction. For example, P2-/P3-$Na_{2/3}Mg_{1/3}Mn_{2/3}O_2$ deliver a large oxygen-redox capacity over 100 mAh/g with large polarization, but exhibit no transition-metal migration (*Nature Chem.* **2018**, *10*, 288-295, *J. Mater. Chem. A* **2019**, *7*, 1491-1498), where O-O dimers could be formed without cation migration. We agree that 'There is no evidence of peroxide formation in these compounds.', but in turn this fact does not deny the formation of O-O dimers in these materials. This scenario 'O-O dimerization without cation migration' is indeed predicted by the DFT calculations in this work (Figure 4).
Concerning two voltage plateaus, the previous ^{23}Na MAS-NMR results clarified the corresponding change in Na^+ arrangement (*Chem. Mater.* **2019**, *31*, 3756-3765). Presumably, the difference in the chemical potential of each Na^+ site gives rise to the two voltage plateaus during the oxygen-redox reaction.
We have added the explanation of the influence of cation migration to oxygen-redox reactions in P7, L22.

'As recently demonstrated using O2-type layered oxides,[23,50] the suppression of cation migration is essential to mitigate the degradation of oxygen-redox cathodes. However, although cation migration should accelerate O-O dimer formation,[16,20,51] the suppression of cation migration alone is not enough to explain nonpolarizing oxygen-redox reaction. For example, P2- and P3-$Na_{2/3}Mg_xMn_{1-x}O_2$ deliver large extra oxygen-redox capacities greater than 100 mAh/g with large polarization but exhibit no transition-metal migration, where O-O dimers could be formed without cation migration..[15,52] This scenario 'O-O dimerization without cation migration' is indeed predicted by the DFT calculations (Figure 4)'

(3) I would suggest to cite the following paper: CrO_2:A Self-Doped Double Exchange Ferromagnet. Phys. Rev. Lett. 80, 4305. The original paper of Zhang-Rice singlet is on sigma anti-bonding (eg*) instead of the pi antibonding (t2g*).

Author reply: We have added the suggested paper as a reference and have mentioned the

difference from the Zhang-Rice singlet in P5, L24.

‘Note that the Zhang-Rice triplet in $Na_{2-x}Mn_3O_7$ arises from π-type direct exchange while the Zhang-Rice singlet originally proposed for superconducting cupper oxides is generated via σ-type direct exchange.[44]’

Reviewer #3 (Remarks to the Author):

(1) This paper describes the reversible anionic redox for Na2Mn3O7 without O-O dimerization, and also provides interesting results for me.

Author reply: Thank you for the positive evaluation.

(2) Absence of O-O dimerization is explained as the origin of smaller voltage hysteresis. However, similar to this work, P3 Na0.6(Li0.2Mn0.8)O2 also shows smaller polarization with possibly anionic redox (Goodenough, 2016 EES). For me, smaller voltage hysteresis is because of the Na chemistry, in which Mn mobility is low compared with the Li chemistry. Mn migration is often used to explain the origin of voltage hysteresis in the literature for the Li chemistry with anionic redox.

Author reply: As recently demonstrated by the research group led by Kisuk Kang (*Nature Mater.* **2020**, *19*, 419-427), the suppression of cation migration is essential for the cycle stability of oxygen-redox cathodes. However, we postulate that, although cation migration may accelerate O-O dimer formation (for example, *Nature Energy* **2020**, *5*, 777-785), the suppression of cation migration alone is not enough to explain nonpolarizing oxygen-redox reaction. For example, P2-/P3-$Na_{2/3}Mg_{1/3}Mn_{2/3}O_2$ deliver a large extra oxygen-redox capacity over 100 mAh/g with large polarization, but exhibit no transition-metal migration (*Nature Chem.* **2018**, *10*, 288-295, *J. Mater. Chem. A* **2019**, *7*, 1491-1498), where O-O dimers could be formed without cation migration. This scenario 'O-O dimerization without cation migration' is indeed predicted by the DFT calculations in this work (Figure 4).

We have added the explanation of the influence of cation migration to oxygen-redox reactions in P7, L23.

'As recently demonstrated using O2-type layered oxides,[23,50] the suppression of cation migration is essential to mitigate the degradation of oxygen-redox cathodes. However, although cation migration should accelerate O-O dimer formation,[16,20,51] the suppression of cation migration alone is not enough to explain nonpolarizing oxygen-redox reaction. For example, P2- and

$P3-Na_{2/3}Mg_xMn_{1-x}O_2$ deliver large extra oxygen-redox capacities greater than 100 mAh/g with large polarization but exhibit no transition-metal migration, where O-O dimers could be formed without cation migration..[15,52] This scenario 'O-O dimerization without cation migration' is indeed predicted by the DFT calculations (Figure 4)'

(3) In addition to the point (2), the concentration of holes formed in this sample is relatively low. A single hole is formed per three Mn ions and seven O ions, and I think that simply lower concentration of holes results in better stability and reversibility (Doublet, 2019 Nature Materials). Indeed, DFT results clearly show the stabilization of O-O dimers as increase in the concentration of holes. This fact inevitably results in that only small reversible capacity is obtained when "nonpolarizing" oxygen redox is used for battery applications.

Author reply: We perfectly agree with this comment. As stated in the main text, the excessive oxidation of oxide ions is an absolute taboo for the reversible oxygen-redox reaction. Therefore, based on this criterion, the oxygen-redox reaction should be an auxiliary mechanism to increase a transition-metal capacity rather than a main charge-compensation mechanism. We have added this explanation with a suggested paper as a reference in P7, L19.

'Based on this criterion, the oxygen-redox reaction should be employed as an auxiliary charge-compensation mechanism under a prudent control rather than as a main charge-compensation mechanism.'

(4) From the assumption of Zhang-Rice triplet formation, in other words, spin interaction between Mn-O occurs, and a hole must be localized in single oxygen. However, from the DFT data, holes seem to be delocalized, and therefore, spin interaction would be not simple as proposed by authors.

Author reply: As pointed out by the reviewer, it is impossible to clearly distinguish between localized and itinerant holes in a strongly correlated electron system such as transition-metal oxides. Indeed, the observed low magnetic moments indicate the itinerant feature of the oxygen hole. We have added the explanation of the competing delocalized/localized features of the

oxygen hole in the main text in P7, L5.

'Note that, in parallel with a localized feature, the oxygen hole has an itinerant feature through the (σ + π) multiorbital interaction. Indeed, the experimentally observed value of *C* for $Na_1Mn_3O_7$ (4.22 cm^3 K mol^{-1}) is slightly lower than that calculated for a localized model (4.75 cm^3 K mol^{-1}) (**Figure 3b**).'

(5) In addition to the point (4), the model used for the magnetic moments seems to be underestimated compared with the experimentally observed values. Any thoughts?

Author reply: We appreciate the valuable comment. As replied to the above comment #4, the itinerant feature of doped hole gives lower experimental values than those calculated for localized models. We have added the explanation of low experimental Curie constants in P7, L4.

'Note that, in parallel with a localized feature, the oxygen hole has an itinerant feature through the (σ + π) multiorbital interaction. Indeed, the experimentally observed value of *C* for $Na_1Mn_3O_7$ (4.22 cm^3 K mol^{-1}) is slightly lower than that calculated for a localized model (4.75 cm^3 K mol^{-1}) (**Figure 3b**).'

Reviewer #2 (Remarks to the Author):

The authors have addressed most of my previous concerns. I would suggest to accept the article in the current version.

However, I would like to point out that the large voltage hysteresis associated with lattice oxygen redox is unlikely originated from the O-O dimerization, and there is no robust experiment evidences to confirm the existence of the so called O-O dimers in most relevant materials. The irreversible cation migration plays an indispensable role (i.e., an ordered layered structure to a more rock-salt like structure, and it is very difficult to move the migrated cations back to the original position during discharge). It is also worth noting that Mg^{2+} and Zn^{2+} are indeed found to migrate to the Na layer in the P2 and P3 type $Na_{2/3}M_{1/3}Mn_{2/3}O_2$ structure, please check the two papers cited. P to O type transition have been observed (either from TEM or XRD), although it is kind of chicken-egg argument about whether it is the cation migration drives the P to O transition or vice versa.

Reviewer #3 (Remarks to the Author):

Well updated on the basis of reviewer comments.

Detailed responses to the review comments with point-to-point basis, including changes highlighted to the manuscript, are listed below.

Reviewer #2 (Remarks to the Author):

(1) The authors have addressed most of my previous concerns. I would suggest to accept the article in the current version. However, I would like to point out that the large voltage hysteresis associated with lattice oxygen redox is unlikely originated from the O-O dimerization, and there is no robust experiment evidences to confirm the existence of the so called O-O dimers in most relevant materials. The irreversible cation migration plays an indispensable role (i.e., an ordered layered structure to a more rock-salt like structure, and it is very difficult to move the migrated cations back to the original position during discharge). It is also worth noting that Mg2+ and Zn2+ are indeed found to migrate to the Na layer in the P2 and P3 type Na2/3M1/3Mn2/3O2 structure, please check the two papers cited. P to O type transition have been observed (either from TEM or XRD), although it is kind of chicken-egg argument about whether it is the cation migration drives the P to O transition or vice versa.

Author reply: Thank you for the valuable comment. We agree that the influence of cation migration and O-O dimerization to O redox is yet to be fully understood. We have added the explanation in P8, L7 as,

'Correlation between cation migration and O-O dimerization involving thermodynamic and kinetic issues, and its influence on the voltage hysteresis is debatable, calling for further studies.'

Reviewer #3 (Remarks to the Author):

(1) Well updated on the basis of reviewer comments.

Author reply: Thank you for reviewing this manuscript.
